# Different strategies, equivalent treatment approaches in terms of mortality in four university hospitals: a retrospective multicenter study of gastroschisis in Finland

**DOI:** 10.1007/s00383-021-04980-5

**Published:** 2021-09-05

**Authors:** Asta Tauriainen, Anna Hyvärinen, Arimatias Raitio, Ulla Sankilampi, Mikko Gärding, Tuomas Tauriainen, Ilkka Helenius, Kari Vanamo

**Affiliations:** 1 Department of Pediatric Surgery, University of Eastern Finland and Kuopio University Hospital, Puijonlaaksontie 2, 70210 Kuopio, Finland; 2 Department of Pediatric Surgery, University of Tampere and Tampere University Hospital, Tampere, Finland; 3 Department of Pediatric Surgery and Orthopedics, University of Turku and Turku University Hospital, Turku, Finland; 4 Department of Pediatrics, Kuopio University Hospital, Kuopio, Finland; 5 Department of Pediatric Surgery, Oulu University Hospital, Oulu, Finland; 6 Department of Surgery, Oulu University Hospital, Oulu, Finland; 7 Department of Orthopedics and Traumatology, University of Helsinki and Helsinki University Hospital, Helsinki, Finland; 8 School of Medicine, University of Eastern Finland, Kuopio, Finland

**Keywords:** Gastroschisis, Mortality, Inter-hospital variability, Surgery

## Abstract

**Purpose:**

Optimal treatment of gastroschisis is not determined. The aim of the present study was to investigate treatment methods of gastroschisis in four university hospitals in Finland.

**Methods:**

The data of neonates with gastroschisis born between 1993 and 2015 were collected. The primary outcomes were short and long-term mortality and the length of stay (LOS). Statistical analyses consisted of uni- and multivariate models.

**Results:**

Total of 154 patients were included (range from 31 to 52 in each hospital). There were no statistically significant differences in mortality or LOS between centers. Significant differences were observed between the hospitals in the duration of mechanical ventilation (*p* = 0.046), time to full enteral nutrition (*p* = 0.043), delay until full defect closure (*p* = 0.003), central line sepsis (*p* = 0.025), abdominal compartment syndrome (*p* = 0.018), number of abdominal operations during initial hospitalization (*p* = 0.000) and follow-up (*p* = 0.000), and ventral hernia at follow-up (*p* = 0.000). In a Cox multivariate analysis, the treating hospital was not associated with mortality.

**Conclusion:**

There were no differences in short or long-term mortality between four university hospitals in Finland. However, some inter-hospital variation in postoperative outcomes was present.

**Level of evidence:**

Level III.

## Introduction

Gastroschisis is a congenital anterior abdominal wall defect with an increasing prevalence worldwide [1–3]. The reported prevalence of gastroschisis varies between 1 and 5 in 10,000 live births [1, 4, 5] and is recently reported to be 1.73 in 10,000 in Finland [1]. The survival rate is over 90% in the western countries [1, 5–8] due to advances in perinatal and neonatal care and early surgical management. Primary closure of the abdominal wall defect is not always possible and various techniques of staged closure have been developed. A commercial spring-loaded silo enables placement at the bedside without the need for operating room or sutures [9–11]. Other types of silos are also used, such as plastic infusion bags [12]. Sutureless umbilical closure technique has been reported to be successful in selected cases [13–15]. Despite the recent advances, the optimal approach to management of infants with gastroschisis remains controversial [16, 17]. The aim of the present study was to investigate treatment methods of gastroschisis in four Finnish university hospitals and to determine, whether the variability across hospitals could be explained by key patient- and hospital level factors, which would be amenable to a protocol-driven approach.

## Materials and methods

### Patients

We conducted a retrospective study of neonates born with gastroschisis between 1st of January 1993 and 31st of December 2015 and treated at neonatal intensive care units and pediatric surgery departments of four university hospitals, Tampere, Turku, Kuopio, and Oulu, Finland. Infants with gastroschisis were identified from the hospital files using the International Classification of Diseases, 9th Revision codes for gastroschisis 756.73 and after the year 1994, ICD-10 code Q79.3. The demographic data, mode of delivery, initial presentation at birth, technique of surgical management, postoperative treatment, complications, short and long term outcomes were collected from the patient records of each hospital. Birth weight and gestational age were obtained from the Finnish Medical Birth Register maintained by the Finnish Institute for Health and Welfare (THL). The study was carried out according to Finnish national and European Union legislation and guidelines. The institutional review board of the university hospitals and the Finnish Institution for Health and Welfare accepted the study (THL/206/5.05.00/2017). The need for patients’ written consent was deemed unnecessary by the institutional review boards as we did not contact the families to conduct this retrospective study. The delivery of all prenatally detected gastroschisis babies was planned to take place in their local university center. All patients included in the present study were treated in the four university hospitals described above.

### Definitions

The birth weight *Z* score was obtained using the contemporary Finnish Birth size reference [18]. Small for gestational age (SGA) was defined as birth weight below 2 standard deviations (SD) [19]. Prematurity was defined as birth before 37th gestation week. Septic infections associated with the peripherally or centrally inserted central lines were defined as positive blood and/or catheter tip cultures combined to clinically septic presentation registered in the patient records. Center status is defined as the treating university hospital. Primary fascial sutured closure was defined as sutured fascia and skin in the primary operation. None of the participating centers used sutureless primary closure with the umbilical cord during the study period. Silo complication was defined as a detached silo or a silo-related hemodynamic complication. Parenteral nutrition was defined as intravenous glucose, amino acids, and/or lipids infusions. The severity of gastroschisis was analyzed with simple/complex categories. Patients with gastroschisis combined to bowel atresia, bowel perforation, bowel necrosis or volvulus were classified as having a complex gastroschisis [20, 21]. The number of gastrointestinal operations (any abdominal operation on general anesthesia, including tucking of silo) and severe gastrointestinal complication (intestinal perforation, any bowel resection, mechanical intestinal obstruction resulting in a laparotomy, abdominal compartment syndrome, and enterocolitis) were defined according to the criteria suggested by Allin et al. [22]. Abdominal compartment syndrome was defined as increasing abdominal distension resulting in a laparotomy with evidence of decreased organ perfusion. Furthermore, intra-abdominal pressures were measured from the urinary bladder and pressures above 20 mmHg were deemed abdominal compartment syndrome. However, the measurement was not used in all cases. The data were divided into five periods according to the patients’ date of birth; 1: 1993–1995, 2: 1996–2000, 3: 2001–2005, 4: 2006–2010, 5: 2011–2015.

### Closure techniques

#### Primary fascial sutured closure

Primary fascial sutured closure was generally preferred in all participating centers whenever possible. Intra-abdominal pressure was regularly monitored from the bladder, stomach, or central line during and after the procedure. A manual detraction or sharp incision of the abdominal wall was performed in cases where bowel reduction was otherwise impossible. Bowel decompression from meconium was achieved with a nasogastric tube or a small enterotomy (*n* = 5). Any bowel diverticulums encountered during the operation were resected based on the surgeon’s preference. Fascial defect closure was performed in a single- or double-layer fashion.

#### Staged closure

Staged closure consists of any preformed silo, roller-silo [23] or customized silo system that gradually repositions the bowel into the abdominal cavity before final defect closure. Preformed spring-loaded silos were used in our cohort after the year 2003. The silo is placed over the protruded viscera under the fascial defect. No sutures are generally required; however, in 7 cases the spring-loaded silo edges were sutured. In the Gore–Tex silo technique, one or two Gore–Tex patches were sutured to the edges of the defect. Staged reduction was achieved by gradually suturing the Gore–Tex patches closer to each other in the midline. The roller silo system consists of a metallic rod attached to the silo and hanged with two pairs of runners from the ceiling of the crib. The rod is left to turn freely on its own weight and squeeze the contents of the silo slowly into the abdominal cavity.

#### Secondary closure

Secondary closure with a patch was used as a last option, if staged primary closure, i.e., silo treatment failed to bring the edges together, or sometimes during the initial operation, if the patient was suspected to require no additional operations and fascial closure was not otherwise possible.

### Outcomes

The primary outcomes were the short and long-term mortality and the length of stay (LOS), and secondary outcomes were the duration of mechanical ventilation, time to abdominal wall closure, central line sepsis, number of operations, number of gastrointestinal complications, and the time to full enteral nutrition. These outcomes were compared between the hospitals (A, B, C, and D) and between the five time periods.

### Statistical analysis

Statistical analyses were performed with SPSS software (version 25.0, IBM Corporation, New York, USA). No attempt to replace missing values was made. Categorical variables are reported as counts and percentages. Continuous variables are reported as medians and interquartile ranges (IQR). Chi-square and Fischer’s exact tests were used for the analyses of categorical variables whereas Mann–Whitney *U* test and Kruskal–Wallis test were used to analyze continuous data. The mortality in different institutions were compared with the Funnel plot analysis. A Cox regression analysis was undertaken to evaluate the effect of treating hospital on mortality. All statistical tests, except the funnel plot analysis, were performed as two-tailed and a *p* value ≤ 0.05 represented statistical significance.

## Results

A total of 155 newborns with gastroschisis born between 1st of January 1993 and 31st of December 2015 were identified. One neonate was excluded due to missing records, which resulted in a total of 154 patients. Number of treated patients in the four participating university hospitals ranged from 31 to 52. The patient baseline data, operative variables, and outcomes of gastroschisis treatment in the participating hospitals are presented in Table 1. There were 86 male (55.8%) and 68 female infants in the whole cohort. The median birth weight and gestational age in the overall series were 2470 g and 36.9 weeks, respectively. In hospital C, there were more premature infants when compared with all other centers 67.3 vs 45.5% (*p* = 0.011, Chi-Square test) and their birth weight (*p* = 0.041, Mann–Whitney *U* test) was smaller than in the other three participating hospitals. The median birth weights were 2320 g in hospital C vs 2605 g, 2460 g, and 2485 g in hospitals A, B, and D, respectively. Vaginal delivery rate in the overall series was 31.2 percent with a range from 7.7 to 64.5% (*p* = 0.000) between the centers.

**Table 1:** Comparison of the treating hospitals in newborns with gastroschisis

Variables	Overall series *n* = 154	Hospital A *n* = 36	Hospital B *n* = 35	Hospital C *n* = 52	Hospital D *n* = 31	*p* value
Baseline data						
Gestational age (weeks)	36.9 ± 2.4 [29.3–40.3]	37.4 ± 2.1 [33.9–40.3]	36.9 ± 2.5 [33.3–38.9]	36.4 ± 1.7 [30.4–39.9]	37.3 ± 2.8 [29.3–40.0]	0.024
Birth weight (g)	2470 ± 709 [1000–4960]	2605 ± 590 [1800–4830]	2460 ± 480 [1425–4960]	2318 ± 885 [1375–4090]	2485 ± 600 [1000–4050]	0.040
Vaginal birth	48 (31.2)	10 (27.8)	14 (40.0)	4 (7.7)	20 (64.5)	0.000
Prematurity	82 (53.2)	16 (44.4)	19 (54.3)	35 (67.3)	12 (38.7)	0.048
SGA	56 (36.4)	11 (30.6)	14 (40.0)	30 (57.7)	22 (71.0)	0.524
Gender: male	86 (55.8)	23 (63.9)	19 (54.3)	28 (53.8)	16 (51.6)	0.732
Prenatal diagnose known	130 (87.8)	33 (91.7)	25 (71.4)	45 (97.8)	27 (87.1)	0.003
Procedures under general anesthesia						
Primary visceral reduction and sutured fascial closure without patch	87 (56.5)	23 (63.9)	22 (62.9)	23 (44.8)	19 (1.3)	0.183
Primary visceral reduction and sutured fascial closure with patch	7 (4.5)	0 (0.0)	1 (2.9)	5 (9.6)	1 (3.2)	0.161
Staged visceral reduction (silo) and fascial closure	60 (39.0)	13 (36.1)	12 (34.3)	24 (46.2)	11 (35.5)	0.862
Staged visceral reduction (silo) and secondary fascial closure with patch^*β*^	5 (3.2)	2 (5.6)	1 (2.9)	2 (3.8)	0 (0.0)	0.631
Operative variables						
Days to closure (silo treatment excluding deceased *n* = 56)	7.0 ± 6.0 [1.0–25.0]	3.0 ± 10.0 [1.0–13.9]	7.0 ± 4.5 [3.0–20.0]	5.5 ± 3.0 [1.0–10.0]	12.0 ± 8.0 [7.0–25.0]	0.001
Complex gastroschisis	21 (13.6)	2 (5.6)	4 (11.4)	12 (23.1)	3 (9.7)	0.090
Outcome measures						
Mechanical ventilator time (days)	4.0 ± 6.0 [0.0–83.0]	3.0 ± 3.0 [0.0–26.0]	6.0 ± 6.0 [1.0–21.0]	3.0 ± 5.0 [0.0–23.0]	3.0 ± 10.0 [0.0–83.0]	0.046
Mechanical ventilator time (days) excluding deceased	3.5 ± 5.0 [0.0–36.0]	3.0 ± 3.0 [0.0–26.0]	6.0 ± 5.8 [1.0–21.0]	3.0 ± 4.8 [0.0–13.0]	3.0 ± 10.0 [0.0–36.0]	0.049
Abdominal compartment	5 (3.2)	0 (0)	0 (0)	5 (9.6)	0 (0)	0.018
Short bowel syndrome	7 (4.5)	2 (5.6)	0 (0)	5 (9.6)	0 (0)	0.101
Necrotising entrocolitis	2 (1.3)	1 (2.8)	0 (0)	1 (1.9)	0 (0)	0.655
Wound infection	22 (14.3)	4 (11.1)	8 (23.5)	5 (9.6)	5 (16.7)	0.297
Central line sepsis	20 (13.0)	8 (22.2)	7 (21.2)	5 (10.2)	0 (0)	0.025
Time to full enteral nutrition (days)	21.0 ± 16.0 [1.0–1869.0]	18.5 ± 15.5 [9.0–639.0]	16.0 ± 13.0 [5.0–148.0]	25.0 ± 17.0 [1.0–1869.0]	23.0 ± 13.0 [10.0–47.0]	0.043
Time to full enteral nutrition (days) excluding deceased	21.0 ± 16.0 [5.0–1869]	18.0 ± 16.0 [9.0–639.0]	16.0 ± 13.5 [5.0–148.0]	27.0 ± 16.5 [13.0–1869.0]	23.0 ± 11.8 [10.0–47.0]	0.001
Time to full enteral nutrition excluding short bowel syndrome patients and deceased (days)	21.0 ± 14.0 [5.0–148.0]	17.5 ± 15.3 [9.0–70.0]	16.0 ± 13.5 [5.0–148.0]	25.0 ± 13.5 [13.0–95.0]	23.0 ± 11.8 [10.0–47.0]	0.007
Time to first enteral nutrition (days)	7.0 ± 7.3 [1.0–39.0]	5.0 ± 5.8 [2.0–34.0]	8.5 ± 5.8 [1.0–9.0]	6.0 ± 6.0 [2.0–38.0]	6.0 ± 12.0 [2.0–39.0]	0.277
Number of abdominal operations during the initial hospitalization^*α*^	1.0 ± 1.0 [1.0–6.0]	1.0 ± 1.0 [1.0–6.0]	1.0 ± 2.0 [1.0–6.0]	2.0 ± 1.0 [1.0–4.0]	1.0 ± 1.0 [1.0–6.0]	0.000
Severe complication during the initial hospitalization	23 (15.0)	4 (11.1)	5 (14.7)	12 (23.1)	2 (6.5)	0.183
Length of stay (days)	27.0 ± 18.5 [1.0–372.0]	21.5 ± 22.3 [10.0–372]	26.0 ± 15.0 [7.0–149.0]	28.0 ± 16.0 [1.0–345.0]	27.0 ± 20.0 [10.0–166.0]	0.400
Length of stay (days) excluding deceased	27.0 ± 17.0 [7.0–372]	21.0 ± 21.0 [10.0–372]	25.5 ± 14.8 [7.0–149.0]	29.0 ± 15.3 [18.0–345.0]	27.0 ± 17.8 [12.0–166.0]	0.053
Length of stay excluding deceased and hospital transfer at discharge	27.0 ± 17.3 [7.0–345.0]	21.0 ± 18.0 [11.0–73.0]	26.0 ± 14.0 [7.0–120.0]	29.0 ± 16.5 [19.0–345.0]	27.0 ± 17.0 [14.0–166.0]	0.058
Ventral hernia at follow-up	22 (14.3)	0 (0.0)	16 (45.7)	2 (3.8)	4 (12.9)	0.000
Number of abdominal operations at follow-up^*α*^	0.0 ± 1.0 [0.0–4.0]	0.0 ± 1.0 [0.0–2.0]	1.0 ± 1.0 [0.0–4.0]	0.0 ± 1.0 [0.0–1.0]	0.0 ± 0.0 [0.0–4.0]	0.000
Severe complication during follow-up	16 (10.4)	4 (11.1)	4 (11.4)	6 (11.5)	2 (6.5)	0.885
Overall mortality	14 (9.1)	1 (2.8)	3 (8.6)	7 (13.5)	3 (9.7)	0.397
Neonatal mortality within the first 28 days	8 (5.2)	0 (0)	2 (5.7)	5 (9.6)	1 (3.2)	0.231
Late mortality	6 (3.9)	1 (2.8)	1 (2.9)	2 (3.8)	2 (6.5)	0.858

Nominal variables are presented as counts and percentages. Continuous variables as median and interquartile range in addition to range in brackets

*SGA* small for gestational age

^α^Performed under general anesthesia

^β^Is included in staged visceral reduction (silo) and fascial closure

Table 2 describes the differences of key demographic and outcome variables between our chosen eras. Vaginal delivery rate varied between 19.6 and 41.2% over the years. Planned cesarean section was more common if the diagnosis of gastroschisis was made prenatally (*p* = 0.001, Chi Square). Significant differences were observed in the prenatal detection rate of gastroschisis which showed an increasing trend towards the end of our study period from 87.5% in 1993–1995, 68.2% in 1996–2000, 80.6% in 2001–2005, 93.5% in 2006–2010 to 97.6% in 2011–2015, *p* = 0.006. Overall, the rate of prenatal diagnosis of gastroschisis varied between 71.4 and 97.8% in our participating centers. Moreover, the rate of primary visceral reduction and sutured closure without a patch was found to decrease significantly towards the end of our study period, whereas treatment with a silo increased, *p* = 0.006 and *p* = 0.036, respectively.

**Table 2:** Differences in key demographic and outcome variables during our five time frames of the study period

Time frames	Time frame 1 1993–1995	Time frame 2 1996–2000	Time frame 3 2001–2005	Time frame 4 2006–2010	Time frame 5 2011–2015	*p* value
Number of patients	9	24	34	46	41	
Vaginal birth	4 (44.4)	7 (29.2)	14 (41.2)	9 (19.6)	14 (32.1)	0.246
Prenatal diagnose known	7 (87.5)	15 (68.2)	25 (80.6)	43 (93.5)	40 (97.6)	0.006
Prematurity	4 (44.4)	13 (54.2)	15 (44.1)	28 (60.9)	22 (53.7)	0.644
Primary visceral reduction and sutured fascial closure without patch	7 (77.8)	16 (66.7)	22 (64.7)	27 (58.7)	15 (36.6)	0.036
Primary visceral reduction and sutured fascial closure with patch	1 (11.1)	N/A	2 (5.9)	4 (8.9)	N/A	N/A
Staged visceral reduction (silo) and fascial closure	1 (11.1)	8 (33.3)	10 (29.4)	15 (32.6)	26 (63.4)	0.004
Staged visceral reduction (silo) and secondary fascial closure with patch^*β*^	N/A	1 (4.2)	1 (2.9)	N/A	3 (7.3)	N/A
Days to closure (silo treatment excluding deceased *n* = 56)	7.0 [7.0–7.0]	9.0 [7.0–14.0]	10.0 [3.0–25.0]	8.0 [2.0–12.0]	6.0 [1.0–20.0]	0.081
Complex gastroschisis	2 (22.2)	3 (12.5)	5 (14.7)	6 (13.0)	5 (12.2)	0.950
Mechanical ventilator time (days)	6.0 [3.0–13.0]	5.0 [1.0–20.0]	4.0 [1.0–36.0]	3.0 [0.0–83.0]	4.0 [0.0–21.0]	0.277
Abdominal compartment	0 (0)	1 (4.3)	2 (5.9)	2 (4.3)	0 (0)	0.613
Central line sepsis	2 (22.2)	4 (17.4)	4 (12.5)	5 (11.4)	5 (12.2)	0.886
Time to full enteral nutrition excluding short bowel syndrome patients and deceased (days)	17.5 [13.0–39.0]	16.0 [10.0–60.0]	18.5 [5.0–70.0]	21.5 [9.0–53.0]	22.0 [9.0–148.0]	0.808
Time to first enteral nutrition (days)	10.0 [3.0–14.0]	8.5 [3.0–39.0]	6.0 [2.0–34.0]	7.0 [2.0–38.0]	6.0 [1.0–19.0]	0.181
Number of abdominal operations during the initial hospitalization^*α*^	2.0 [1.0–6.0]	1.0 [1.0–6.0]	1.0 [1.0–5.0]	1.0 [1.0–6.0]	2.0 [1.0–6.0]	0.247
Severe complication during the initial hospitalization	2 (22.2)	5 (21.7)	6 (17.6)	7 (15.2)	3 (7.3)	0.514
Length of stay excluding deceased and hospital transfer at discharge	33.5 [19.0–55.0]	27.0 [14.0–166.0]	28.0 [7.0–345.0]	28.5 [14.0–255.0]	25.5[11.0–100.0]	0.734
Number of abdominal operations at follow-up^α^	0.0 [0.0–2.0]	0.0 [0.0–2.0]	0.0 [0.0–4.0]	0.0 [0.0–4.0]	0.0 [0.0–1.0]	0.528
Severe complication during follow-up	0 (0)	3 (12.5)	2 (5.9)	7 (15.2)	4 (9.8)	0.546
Overall mortality	0 (0)	4 (16.7)	3 (8.8)	7 (15.2)	0 (0)	0.067
Neonatal mortality within the first 28 days	0 (0)	2 (8.3)	3 (8.8)	3 (6.5)	0 (0)	0.368
Late mortality	0 (0)	2 (8.3)	0 (0)	4 (8.7)	0 (0)	0.112

Nominal variables are presented as counts and percentages. Continuous variables as median and range in brackets

The time frames are 1: 1st of Jan 1993–31st of Dec 1995, 2: 1st of Jan 1996–31st of Dec 2000, 3: 1st of Jan 2001–31st of Dec 2005, 4: 1st of Jan 2006–31st of Dec 2010, 5: 1st of Jan 2011–31st of Dec 2015

*N/A* not available

^α^Performed under general anesthesia

Immediately after birth, the intestines were protected with plastic film (in 64.3% of the cases) or with moist bandage to avoid temperature loss and peritoneal dehydration. The severity of the gastroschisis, based on the rate of complex gastroschisis did not differ significantly between the hospitals (Table 1). The rates of different fascial closure methods were similar between each participating hospital. Median days to full defect closure with delayed techniques was 7.0 in overall series, excluding four patients who deceased before abdominal closure. Surprisingly, in hospital D, time to closure was significantly longer (12.0 days) than in all other centers (3.0, 7.0 and 5.5 days in hospitals A, B, and C, respectively) (*p* = 0.001, Kruskal–Wallis test).

Overall median mechanical ventilator time was 4.0 days ranging from 0 to 83 days. A significant difference between the centers was observed (*p* = 0.046). The longest mechanical ventilator time was observed in hospital B, 6.0 days vs 3.0, in all hospitals A, C, and D, respectively. The overall median time to full enteral nutrition was 21.0 days. Time to full enteral nutrition was the longest in hospital C (median 25.0 days), and there was a significant difference between the centers (*p* = 0.043). The time to full enteral nutrition in hospitals A, B, and D were 18.5, 16.0, and 23.0 days, respectively. The overall median length of stay was 27.0 days, and there were no statistically significant differences between the centers.

The incidence of central line associated sepsis ranged from 0.0 to 22.2%, a significant difference between the centers was observed (*p* = 0.025). Interestingly, the number of gastrointestinal operations during and after the initial hospitalization period differed significantly (*p* = 0.000 in both), however, the incidence of severe gastrointestinal complications did not differ between the centers during or after the primary hospitalization period (*p* = 0.183 and *p* = 0.885, respectively). The median number of gastrointestinal operations during the initial hospitalization were 1, 1, 2, and 1 and after the initial hospitalization 0, 1, 0, and 0 in hospitals A, B, C, and D, respectively.

Fascial patches were used in 7 cases at the primary operation. Five patients received a fascial patch at the end of a silo treatment. In some cases where fascial closure was impossible, only the skin was sutured in the primary operation (*n* = 3). Seventy patients (45.5%) required sharp fascial incision with scissors or knife to facilitate bowel reduction into the abdomen. Sharp fascial incision was not associated with the need for a patch, 5 (7.4%) patients in incision group and 6 (8.1%) patients in no incision group, *p* = 0.866. However, sharp fascial incision was associated with the need for a silo, 34 (48.6%) patients versus 23 (30.7%) patients, *p* = 0.027 respectively. Furthermore, there were significant differences in the rate of ventral hernia repair during the follow-up period between the centers (0,0–45,7%, *p* = 0.000).

**Fig. 1:**
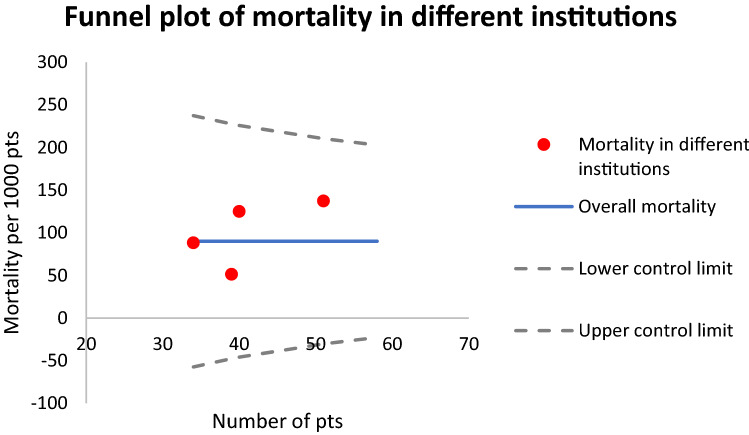
Funnel plot of mortality in different institutions based on the national database and including four Finnish university hospitals. The horizontal axis measures the amount of gastroschisis cases in each institution. The vertical axis measures the mortality expressed as a rate per 1000 cases. The dots mark the mortality per 1000 patients in institutions. The horizontal central line shows the overall mortality, in this case 90.9 per 1000 patients. The dashed lines constitute the funnel. They are the upper and lower 3 SE (standard error) control limits that represent the boundary between ‘normal variation’ and ‘special cause variation’. All institutions are well within the control limits

Number of surviving patients in the present study was 140 (90.9%) during our follow up time (mean 11 years). There were no statistically significant differences between the hospitals regarding patient mortality, although hospital C had relatively higher values of overall mortality (13.5 vs 2.8%, *p* = 0.132) compared with hospital A. In the funnel plot analysis of mortality in the four participating university hospitals in Finland, the mortality rates were well within the control limits (Fig. 1). We analyzed a trend in the prevalence of neonatal, late, and overall mortality at our five time frames, which did not show any statistical differences during our study period.

We performed a comparison of any direct closure (i.e. bowel reduction and fascial closure with or without a patch at the primary operation) to any staged silo closure. In our data, any direct closure was associated with lower mortality. Mortality rate was 5.4%, (5 cases) in the any direct closure group and 15.0% (9 cases) in the silo group, however the result was not statistically significant (*p* = 0.055). With any direct repair, we reported lower median ventilator time, 3 (IQR 2) days versus 8 (IQR 10) days in the silo group (*p* = 0.000). Median time to full enteral nutrition was also shorter in direct closure 17 (IQR 12) days versus 27 (IQR 15) days in the any silo group, (*p* = 0.000). Length of hospital stay was shorter in any direct closure compared with any staged closure, 25 (IQR 14.5) days versus 31 (IQR 22) days (*p* = 0.004), respectively.

Finally, a Cox proportional hazards model was used to assess the impact of treating hospital on the risk of death. The model included the following variables: gestational age, birth weight, Apgar score at 1 min, treatment with silo, relaparotomy for bowel perforation or necrosis, pulmonary hypoplasia, center status, complex gastroschisis, and the period of the treatment. The model variables were adapted from the study by Tauriainen et al. [24]. The treating hospital or the period of the treatment were not associated with mortality in the Cox regression analysis.

## Discussion

The demographic data of the present study are consistent with the literature. When comparing the treating hospitals there were statistically significant differences in patient gestational age, weight, the rate of vaginal delivery, and prematurity. A few large scale, multicenter studies addressing gastroschisis outcomes have been published. Bradnock et al. [25] and Owen et al. [26] present a database of 393 cases from all 28 pediatric surgical centers from the United Kingdom. In the study by Gonzalez et al. [27], 4459 gastroschisis patients from the United States were analyzed; they concluded that significant inter-hospital variability persists due to differences in institutional policies [27].

The incidence of complex gastroschisis has been reported to vary between 10.2 and 11.5% [8, 25, 26], which is close to our finding of 13.6%, however a rate as high as 20.8% has been reported [27]. Our reports ranged between 5.6%, 11.4%, 23.1%, and 9.7% in Hospitals A, B, C, and D, respectively.

Statistically significant differences between our participating hospitals were observed regarding time to full enteral nutrition, mechanical ventilator time, central line sepsis, abdominal compartment syndrome, delay until full defect closure, number of abdominal operations during initial hospitalization and during the follow-up, in addition to the rate of ventral hernia at follow-up. Ventral hernia and the number of abdominal operations during follow-up were the highest in hospital B among our participating centers (45.7% and 1.0 ± 1.0, respectively). In this hospital the fascial and cutaneous defects were sometimes closed as one layer, which may have predisposed the patient for a ventral hernia during growth.

Time to full enteral nutrition in the present study was slightly lower than what has been reported in the literature (median of 21 days). According to Kidd et al. [28] time to full enteral feeds was reported 31 days, however, they excluded patients with complex comorbidities such as intestinal and cardiac anomalies [28]. Raymond and colleagues [29] reported on 566 gastroschisis patients with a median parenteral nutrition time of 27 days. In their study, simple gastroschisis patients had 25 and complex gastroschisis patients 64 days [29]. We speculate that the lower times to full enteral nutrition in the present study could be due to our lower proportion of patients treated with silo closure, our 39.0% as compared to 56.1% in the study by Gonzalez [27]. In the overall series the median mechanical ventilator time excluding deceased patients was 3.5 days, which is in line with the 4–7 days reported in the literature [10, 29, 30].

Several studies have investigated the optimal technique of gastroschisis defect closure and the results remain conflicting. A meta-analysis provided evidence that primary closure was associated with improved outcomes regarding length of stay, ventilator days, and parenteral nutrition duration as compared to silo closure [16]. Silo closure was associated with better clinical outcomes (ventilator days, time to first feed and infection rates) in the studies which selected closure technique randomly or as a temporal shift in practice. Another meta-analysis by Ross et al. [31], including 18 studies, demonstrated no differences in ventilator time, but a longer parenteral nutrition time (mean difference 6.4 days) in infants treated with a preformed silo as compared to alternative treatment strategies. Interestingly, a subgroup analysis of four studies, which reported a routine use of preformed silos revealed that the use of preformed silos was associated with shorter ventilator time (mean difference 2.2 days) when compared with other strategies [31]. Indeed, the management of simple gastroschisis can be performed suturelessly without the need for surgical defect closure requiring general anesthesia and a ventilator, which could be beneficial especially in low-resource settings as suggested by Wright and colleagues [32].

Fraser and colleagues [15] demonstrated in their retrospective study with 315 neonates with uncomplicated gastroschisis that primary sutureless closure was associated with shorter mechanical ventilator times (0 vs 3 days) and less anesthetics use when compared with primary sutured closure [15]. Chesley et al. [17] showed in their study of 202 gastroschisis infants that direct fascial closure or sutureless umbilical closure was independently associated with a shorter time in the intensive care unit (*p* < 0.001) and time to initiation of enteral feeding (*p* < 0.01) [17]. A smaller series with 31 gastroschisis infants by McNamara et al. reported that infants with primary reduction with defect closure required shorter duration of parenteral nutrition (21.63 vs 41.83 days) and shorter length of stay (29.13 vs 53.57 days) as compared to staged closure though the results were not statistically significant [33]. The present study is in concordance with these findings, primary closure should be pursued whenever possible. The problem is that in many cases one really does not have a choice between primary closure or a staged procedure, which makes their comparison difficult, even futile.

Hong and colleagues [34] have reported no differences in mortality, sepsis rates and length of stay by center volume (defined as low, medium, or high volume) in their prospectively collected data of 4663 infants with gastroschisis [34]. In Finland, we have a lower incidence of gastroschisis in each participating university hospital as compared to larger centers abroad, and thus it is appropriate to centralize management of the condition to university hospitals, our tertiary care centers. The smaller volume central hospitals do not provide on-call pediatric surgical service or neonatal intensive care. Interestingly, Dubrovsky et al. [35], in their series of 7768 gastroschisis patients, found that centers with different volumes had similar mortality rates [35]. Indeed, the rates of early and late mortality in addition to the length of in-hospital stay did not differ between our participating centers. Considering this evidence and our present findings, we suggest that our current system of treating gastroschisis in Finland is functional and safe, however in the light of our results, some practice policies might be reasonable to undergo re-evaluation.

In a funnel plot, one might argue that a limit of 3 standard errors is too high for an outcome such as mortality, however, the treating center was not found as an independent predictor for mortality in our Cox regression model. Instead, re-laparotomy for perforation or necrosis was found as an independent predictor for mortality in our previous multivariate analysis [24]. We speculate that the first 1–2 years after birth are the most crucial in determining the patients’ survival, since all the deceased patients in our dataset were under 1.4 years of age.

As mortality is nowadays fairy low, more emphasis is given to morbidity. In theory, the best method, when primary reduction and defect closure turns out to be impossible, is the one with the shortest duration of silo reduction, mechanical ventilation, and parenteral nutrition, and the least number of secondary operations, with an appeasing cosmetic result promoting mother and child bonding. Furthermore, many long-term factors such as chronic dysmotility, need for regular hospitalizations and quality of life play a key role in evaluating the results of the management of gastroschisis. A nationwide database and practical co-operation would be imperative to form a national consensus, which would help to achieve these results.

### Limitations

The study was associated with a number of limitations. These include the retrospectively collected data with some missing values and the relatively small sample size in addition to the lack of data on long-term factors as described above. The second major limitation is the lack of data from the fifth, and largest Finnish university hospital in Helsinki. Prospective multicenter studies are required to investigate the optimal management of gastroschisis. Standardized outcome measures for national databases could allow a better comparison of management in Finland.

## Conclusions

There is a notable variability in the presentation and management of gastroschisis patients in four Finnish university hospitals, while the results regarding short- and long-term mortality and length of hospital stay are equal and comparable to international standards. Our data demonstrate that infants with gastroschisis can be managed in relatively low volume centers with acceptable results. Nevertheless, the observed variation calls for a nationwide database and cooperation to guarantee best and equal results.
